# Prevalence and Prognostic Impact of Diabetes Mellitus in Hospitalized COVID-19 Patients: A Monocentric Study From India

**DOI:** 10.7759/cureus.76902

**Published:** 2025-01-04

**Authors:** Nidhi Tripathy, Ashutosh Jain, Jaya Jain

**Affiliations:** 1 Department of Biochemistry, Index Medical College, Hospital & Research Center, Indore, IND; 2 Department of Physiology, Index Medical College, Hospital & Research Center, Indore, IND

**Keywords:** covid-19, diabetes mellitus, disease severity, in-hospital mortality, prevalence, prognosis

## Abstract

Background

COVID-19 has been a fatal pandemic in modern history, with diabetes mellitus (DM) as a common comorbidity. However, data on the effect of DM on Indian COVID-19 patients are still scarce. We aimed to evaluate the prevalence and prognostic impact of DM in COVID-19 patients hospitalized at Index Medical College Hospital and Research Center (IMCHRC), Indore, Madhya Pradesh.

Methods

This is a retrospective monocentric observational study including all consecutive adult COVID-19 patients admitted to our center from July 2021 to March 2022. Data including demographics, clinical features, DM history, laboratory investigations, comorbidities, disease severity, intensive care unit (ICU) admission, invasive fungal infections, multi-organ dysfunction, and death were retrieved from the medical records of the patients and analyzed.

Results

A total of 357 COVID-19 patients (236 males) with a mean age of 52+9.7 years were evaluated. The overall prevalence of DM in the patients was 32% (115/357), comprising of 23% (83/357) cases of pre-existing DM and 9% (32/357) cases of new onset of DM. Vascular comorbidities consisting of hypertension (62% vs 27%; p=0.002), cardiovascular disease (39% vs 21%; p=0.016), and kidney disease (42% vs 27%; p=0.019) were significantly associated with DM. Diabetic versus non-diabetic COVID-19 patients had a higher rate of ICU admission (23% vs 10%; p=0.042), severe disease (43% [49/115] vs 5% [13/242]; p=0.002), acute respiratory distress syndrome (25% vs 7%), secondary infections (30% vs 11%; p=0.033), multi-organ dysfunction (16% vs 9%; p=0.045), and in-hospital mortality (21% [24/115] vs 6% [15/242]; p=0.011).

Conclusions

Our study shows a high prevalence of DM in COVID-19 patients and an adverse effect of DM on the prognosis of COVID-19 patients including increased disease severity and in-hospital mortality.

## Introduction

Coronavirus disease 2019 (COVID-19), a respiratory viral infection caused by the severe acute respiratory syndrome coronavirus 2 (SARS-CoV-2), has been the most devastating pandemic in the modern history [1]. It has been documented to claim around seven million of deaths worldwide, but the actual death toll may be double or even quadruple this official count [2]. India alone has reported to the World Health Organization with more than 5.3 lakhs of confirmed deaths as of December 19, 2023 [3]. Although COVID-19 is presently no longer a global public health emergency, its high mortality still needs further research. Therefore, it is of clinical and public health importance to identify the potential risk factors predisposing COVID-19 patients to severe/critical illness leading to high mortality for improving the outcome of any new wave of COVID-19 or other similar pandemics that we may face in near future.

Diabetes mellitus (DM) has been one of the most common comorbidities overlapping with COVID-19 throughout the pandemic years. In 2021, COVID-19 caused around six million deaths, while DM caused 6.7 million deaths in the same year, showing that DM is a serious risk factor for worse outcomes in COVID-19 patients [4]. The prevalence of DM in COVID-19 patients varied across China and European and North American countries, ranging from 5% to 33.8% [5-8]. Furthermore, SARS-CoV-2 infection has also been reported to aggravate DM or induce new onset of DM in COVID-19 patients [9]. DM is a global disease burden taking shape of a non-communicable pandemic, and India with 77 million DM patients is one of the epicenters of this DM pandemic [10]. An association of DM and COVID-19 was identified initially from India [11], and a recent meta-analysis of 34 Indian studies has reported a significantly higher prevalence of DM and its association with increased risk of mortality in COVID-19 patients [12]. However, robust information from the country on the effect of DM on COVID-19 patients is still scarce.

Therefore, the aim of our study was to determine the prevalence of DM in COVID-19 patients and evaluate the prognostic impact of DM on the outcome of the patients hospitalized at our center.

## Materials and methods

Study design, subjects, and ethics

This is a retrospective single-center observational study including all consecutive adult patients with laboratory-confirmed COVID-19 admitted to Index Medical College Hospital and Research Center (IMCHRC), Indore, Madhya Pradesh, India, from July 2021 to March 2022. The Ethics Committee of IMCHRC, Indore reviewed and approved the study (Approval Code Number: 2020-5-IMP-116 dated November 18, 2020). A waiver of consent of the subjects was granted by the Ethics Committee due to the retrospective nature of the study and the nature of data as part of routine clinical patient care services.

Definitions

COVID-19

COVID-19 was defined by clinical symptoms including fever, cough, dyspnea, and anosmia, and positivity for SARS-CoV-2 RNA in nasal or throat swab samples by reverse-transcription polymerase chain reaction testing as per guidelines of the Indian Council of Medical Research (ICMR, Government of India, www.icmr.gov.in). 

Diabetes

DM status of the patients was defined according to American Diabetes Association (ADA) criteria including glycated hemoglobin (HbA1c) level ≥ 6.5% and fasting blood glucose ≥ 126 mg/dL or random blood glucose level ≥ 200 mg/dL at presentation to the hospital [13]. The patients with HbA1c level ≥ 6.5% and fasting blood glucose ≥ 126 mg/dL with a history of DM were defined as cases of pre-existing DM, while those having elevated HbA1c and fasting blood glucose levels with no history of DM in the past were defined as cases of new onset of DM.

Sample size calculation

The sample size was calculated using the following formula: 

n=Z2P (1-P)/E2

where

n is the sample size, Z is Z-score (1.96 at 95% confidence level), P is the expected prevalence of DM in COVID-19 patients taken as 36% based on a meta-analysis of 34 Indian studies [12], and E is the margin of error (0.05).

Inclusion and exclusion criteria

Adult patients aged > 18 years having a laboratory-confirmed diagnosis of COVID-19 using reverse transcriptase-polymerase chain reaction assay for SARS-CoV-2 RNA in nasal or throat swab samples as per guidelines of the Indian Council of Medical Research (ICMR, Government of India, www.icmr.gov.in) were included in the study. Patients having uncertain diagnosis of COVID-19, age < 18 years, steroid pre-treatment, chronic organ injury or failure, pregnancy, or lack of relevant clinical and laboratory data were excluded from the study.

Data collection and analysis

A format of Microsoft Excel file was designed for systematic data collection. After assigning a specific code to each patient, data on demographics, clinical symptoms, underlying comorbidities, laboratory results, prognostic factors, and clinical outcomes were collected from the medical records of the hospitalized patients with confirmed COVID-19 diagnosis. The data collection within the first day of admission included demographics, clinical features, laboratory results, diabetic history, treatment status, and pre-existing comorbidities. The relevant laboratory assessment at admission included fasting blood glucose (FBG), HbA1c, complete blood count, lactate dehydrogenase (LDH), serum ferritin, procalcitonin, and D-dimer. The data collected during the course of hospital stay of the patients included the disease severity, admission to intensive care unit (ICU), clinical complications including acute respiratory distress syndrome (ARDS), multi-organ dysfunction, and secondary infections, and death. 

The prevalence of DM was calculated as percentage of the included patients having the disease. The demographic features, clinical features, laboratory findings, underlying comorbidities, and various risk factors including disease severity, requirement of ICU admission, development of ARDS and multi-organ dysfunction, occurrence of secondary bacterial and fungal infections, and mortality in diabetic and non-diabetic patients were compared to evaluate impact of DM on the prognosis of COVID-19 patients.

Statistical analysis

Data were computed and analyzed using SPSS Version 22.0 (IBM Corp., Armonk, NY, USA). Variables were expressed as the mean ± SD for data of normal distribution. Depending on whether the data were parametric or non-parametric, appropriate statistical tests of significance including Student’s t-test, Mann-Whitney U test, or chi-square test were applied. A p-value of <0.05 was considered to be statistically significant.

## Results

Selected patients

**Figure 1:**
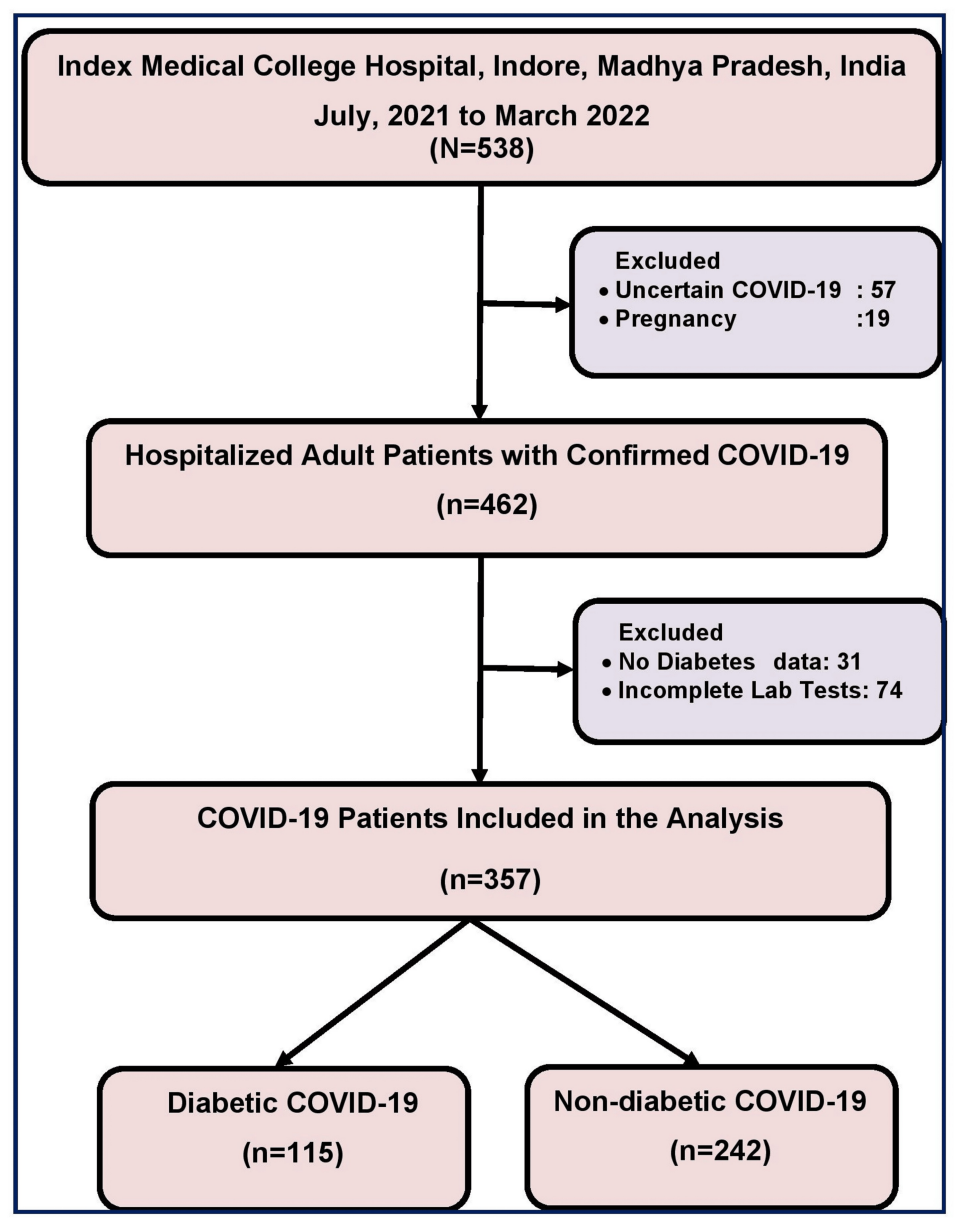
Flow chart showing selection of COVID-19 patients included in the study.

Out of 538 total screened patients, we excluded 57 patients having uncertain diagnosis of COVID-19 and 19 patients having pregnancy. We further excluded 31 patients having no record of DM investigation within 72 hours of admission or no information about pre-existing DM and 74 patients having no relevant laboratory tests. Thus, a total of 357 COVID-19 patients were finally included in the study. Of these, 115 were diabetic and 242 were non-diabetic COVID-19 patients (Figure 1).

Prevalence of DM

Based on the history of DM, HbA1c levels, and admission hyperglycemia, the overall prevalence of DM among the included patients was 32% (115/357), which consisted of 23% (83/357) pre-existing DM (i.e., hyperglycemia with a history of DM) and 9% (32/357) of new onset of DM (i.e., hyperglycemia without diabetic history). Thus, most of our diabetic patients (72%; 83/115) had pre-existing DM, while around one-fourth of these (28%; 32/115) had new onset of DM (Table 1).

**Table 1:** Prevalence of DM in COVID-19 patients DM, diabetes mellitus; COVID-19, coronavirus disease 2019

DM	Prevalence (%) in COVID-19 patients (n=357)
Pre-existing DM	23% ((83/357)
New-onset DM	9% (32/357)
Overall (pre-existing + new onset) DM	32% (115/357)

Baseline characteristics of diabetic and non-diabetic COVID-19 patients

The study population consisted of 357 patients, of whom 236 were males. The mean age of the patients was 52+ 9.7 years. Based on the presence of DM, they were grouped into diabetic (n=115) and non-diabetic (n=242) COVID-19 patients. There was no difference in demographics and clinical features between diabetics and non-diabetics except older age (56.8 + 12.1 vs 45.7+ 16.5; p=0.002) and higher episodes of fever (80% [92/115] vs 66% [161/242]; p=0.032). Among laboratory feature, diabetics as compared to non-diabetics had higher levels of C-reactive protein (CRP) (269.2+ 35.1 vs 49.8 + 31.4 mg/L; p= 0.032), ferritin (407.9 + 40.1 vs 248.7 + 42.3 ng/mL; p=0.016), D-dimer (1.81+5.4 vs 0.76+2.9 μg/mL; p=0.031), leukocyte count (11.23 vs 7.93 x10^3^/μL; p=0.041), and neutrophil count (10.88 vs 7.11 x10^3^/μL; p=0.033), but lower lymphocyte count (0.67 vs 1.18 x10^3^/μL; p=0.004). Among COVID-19 comorbidities, hypertension (62% [71/115] vs 27% [65/242]; p=0.002), cardiovascular disease (39% [45/115] vs 21% [51/242]; p=0.016), and kidney disease (42% [48/115] vs 27% [65/242]; p=0.019) were significantly associated with DM (Table 2).

**Table 2:** Baseline characteristics of diabetic and non-diabetic COVID-19 patients at admission Data are expressed as n (%) unless otherwise specified. The p-values reflect comparisons of diabetic versus non-diabetic COVID-19 patients. CRP, C-reactive protein; FBG, fasting blood glucose; HbA1c, glycated hemoglobin; LDH, lactate dehydrogenase; TLC, total leukocyte count

Characteristics	Total Patients (N=357)	Diabetic (n=115)	Non-diabetic (n=242)	p-Value
Demographics				
Age in years (mean ± SD)	52 ± 9.7	56.8 ± 12.1	45.7± 16.5	0.002
Males	236 (66)	69 (60)	167 (69)	ns
Clinical features				
Fever	253 (71)	92 (80)	161 (66)	0.032
Cough	239 (67)	71 (62)	168 (69)	ns
Headache	132 (37)	40 (35)	92 (38)	ns
Sore throat	83 (23)	22 (19)	61 (25)	ns
Dyspnea	121 (34)	43 (37)	78 (32)	ns
Loss of taste/smell	53 (15)	12 (10)	41 (17)	ns
Nausea	22 (6)	9 (7)	13 (5)	ns
Diarrhea	34 (9)	11 (9)	23 (9)	ns
Fatigue	133 (37)	46 (40)	87 (36)	ns
Abdominal pain	19 (5)	7 (6)	12 (4)	ns
Chest pain	89 (25)	35 (30)	40 (22)	ns
Laboratory features				
TLC (reference range 4.0-10.0 x 10^3^/μL)	7.86 (6.53-12.31)	11.23(7.11-12.31)	7.93 (6.53-8.29)	0.041
Neutrophil count (reference range: 1.8-6.5 x 10^3^/μL)	6.51 (4.12-16.91)	10.88 (6.51-16.91)	7.11 (4.12-10.21)	0.033
Lymphocyte count (reference range: 1.1-3.2 x 10^3^/μL)	0.91 (0.66-1.25)	0.67(0.66-0.87)	1.18 (0.89-1.25)	0.004
Platelet count (reference range: 150-350 x 10^3^/μL)	244(136-463)	256 (151-463).	231 (136–288)	ns
CRP (reference range: <5.0 mg/L), mean ± SD	151.7 ± 94.9	269.2± 35.1	49.8 ± 31.4	0.032
LDH (reference range: 135‑214 U/L), mean ± SD	272.3±33.1	388.2 ± 40.4	330.5 ± 16.4	ns
Ferritin (reference range: 23.9-336.2 ng/mL), mean ± SD	337.8 ±38.9	407.9 + 40.1	248.7 + 42.3	0.016
Procalcitonin (reference range: <0.5ng/mL), mean ± SD	0.20+1.7	0.31±2.7	0.11±1.3	ns
D-dimer (reference range: <0.5 μg/mL), mean ± SD	1.61±3.2	1.81±5.4	0.76±2.9	0.031
FBG (reference range: 65-99 mg/dL), mean ± SD	132 ±13.2	188 ±23.5	102 ±11.8	0.002
HbA1c (reference range: 4.0-5.6 %), mean ± SD	7.1 ± 1.7	8.3 ± 1.6	5.2 ± 0.7	0.015
Diabetic history	83 (23)	83 (72)	NA	NA
Comorbidities				
Any	202 (57)	73 (63)	129 (53)	ns
Hypertension	136 (38)	71 (62)	65 (27)	0.002
Respiratory disease	29 (8)	11 (9)	18 (7)	ns
Cardiovascular disease	96 (27)	45 (39)	51 (21)	0.016
Cerebrovascular disease	34 (9)	13 (11)	21 (9)	ns
Kidney disease	113 (32)	48 (42)	65 (27)	0.019
Liver disease	19 (5)	6 (5)	13 (5)	ns

Prognosis of diabetic and non-diabetic COVID-19 patients

**Figure 2:**
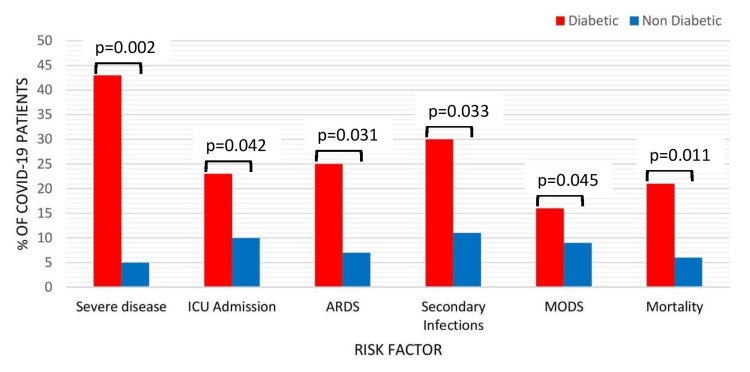
Prognostic risk factors in patients with diabetic and non-diabetic COVID-19 patients ARDS, acute respiratory distress syndrome; ICU, intensive care unit; MODS, multi-organ dysfunction syndrome

We studied disease severity, ICU admission requirement, ARDS, secondary infections, multi-organ dysfunction or failure, and mortality rate as prognostic factors between diabetic and non-diabetic COVID-19 patients. It was observed that diabetic as compared to non-diabetic COVID-19 patients had higher rate of severe disease (43% [49/115] vs 5% [13/242], p=0.002), ICU admission (23% [27/115] vs 10% [24/242]; p=0.042), ARDS (25% [29/115] vs 7% [18/242]; p=0.032), secondary infections (30% [35/115] vs 11% [29/242], p=0.033), multi-organ dysfunction (16% [19/115] vs 9% [22/242], p=0.045), and in-hospital mortality (21% [24/115] vs 6% [15/242], p=0.011) (Figure 2).

## Discussion

Our study shows that DM was present in 32% of the hospitalized COVID-19 patients, and diabetic COVID-19 patients had a worse prognosis than non-diabetic ones. The comorbidities of hypertension, cardiovascular disease, and kidney disease were significantly associated with diabetic COVID-19 patients, and these patients had elevated levels of inflammatory biomarkers including CRP, ferritin, and D-dimer, and high leukocyte and neutrophil counts but low lymphocyte count. The DM in COVID-19 patients was associated with increased rate of ICU admission, severe disease, secondary infections, ARDS, multi-organ dysfunction, and in-hospital mortality. To the best of our knowledge, this is the first study from India showing a robust impact of DM and its associated risk factors on the prognosis of COVID-19 patients.

DM is a global disease burden taking shape of a non-communicable pandemic, and it is one of the most frequently reported comorbidities in COVID-19 patients [14]. According to a recent meta-analysis, the global pooled prevalence of DM was 21.4% in hospitalized COVID-19 patients, among whom the prevalence of DM was 28.9% in severe COVID-19 and 34.6% in those who died of COVID-19 [15]. Similarly, observational studies from China and European and North American countries have reported the prevalence of DM varying from 5% to 33.8% [5-8]. We observed an overall prevalence of 32% of DM in hospitalized COVID-19 patients, which is within the range reported in other countries of the world. In India, the pooled prevalence of DM has been reported to be 17.7%, ranging from 4% to 68% by a recent meta-analysis of 34 Indian studies [12]. In addition, several observational studies from India have reported a prevalence of DM ranging from 5.5% to 63.7% in hospitalized COVID-19 patients [16-18]. These studies from India are in agreement with our data on the prevalence of DM in COVID-19. In our study, 9% of the patients had new onset of DM. Similarly, there are two Indian studies reporting new onset of DM (i.e., newly detected hyperglycemia at admission) in 5.5% and 12.7% of hospitalized COVID-19 patients, supporting our observation [19,20]. A recent meta-analysis on studies from other countries reporting significantly elevated risk of DM in patients with COVID-19 as compared with non-COVID-19 controls further lends support to our observation of new-onset of DM by SARS-CoV-2 infection [21]. Whether this new-onset DM persists permanently in the patients is presently not known, as the long-term follow-up of these patients is limited.

A precise mechanism of COVID-19-induced DM is not clear. It has been shown that angiotensin-converting enzyme 2, transmembrane serine protease 2, neuropilin-1, and transferrin receptor, the main receptors for SARS-CoV-2 entry into human cells, are highly expressed on pancreatic β-cells. The SARS-CoV-2 can directly infect β-cells through binding to these receptors and induce β-cell damage, resulting in impaired insulin secretion, altered glucose metabolism, and hyperglycemia. In addition, insulin deficiency and/or insulin resistance caused by local inflammation and infiltration of immune cells to the pancreas could be another probable explanation for the development of new DM in COVID-19 patients [21,22]. Our observation of new-onset DM in the patients also suggests that like SARS-CoV-2, several other viral infections may be an important etiological trigger of DM in the general population, particularly in developing countries like India, which have a high prevalence of this disease.

DM is not a single isolated disease but rather complex metabolic syndrome of hyperglycemia associated with several micro- and macro-vascular complications adversely affecting multiple organs and systems. In the present study, we have observed a significantly higher association of vascular comorbidities of hypertension, and cardiovascular and kidney diseases with diabetic as compared to non-diabetic COVID-19 patients. Two Indian studies reported a high prevalence of these vascular comorbidities in COVID-19 patients but they have not evaluated their association with DM [19]. The studies from India and other countries reporting hypertension, cardiovascular disease, and kidney disease as major complications associated with DM support the findings of our study [19,23,24]. Our laboratory findings revealed that diabetic COVID-19 patients also had high levels of inflammatory biomarkers including CRP, ferritin, and D-dimer, neutrophilic leukocytosis, and lymphopenia, corroborating with studies reported in the literature [25,26]. These observations of our study collectively suggest that DM greatly increases the risks of vascular complications and systemic inflammation, increasing the severity of disease in diabetic COVID-19 patients.

In order to evaluate the impact of DM on the prognosis of the disease, we compared the association of disease severity, ICU admission requirement, ARDS, secondary infections, multi-organ dysfunction or failure, and rate of mortality with diabetic and non-diabetic COVID-19 patients. We observed a significantly greater association of all these prognostic factors with diabetic as compared to non-diabetic COVID-19 patients, suggesting a negative impact of DM on the outcome of the disease. There are only three Indian studies reporting outcomes of DM on severity and mortality of COVID-19 patients. One study reported a disease severity of 49%, consistent with our study, but a mortality rate of 52% in diabetic COVID-19 patients, which is substantially higher than that in our study [20]. The second study reported comparable mortality rate in diabetic and non-diabetic patients but significantly higher mortality in hyperglycemic COVID-19 patients. This study also observed that after adjusting for age and comorbidities, hyperglycemia at admission was an independent risk factor of mortality in the patients [16]. The third Indian study showed the risk of ICU admission in diabetic COVID-19 patients similar to our study but a lower disease severity and mortality than our study [18]. Although a precise reason for these differences between our and these two Indian studies is not clear, it may be due to differences in the degree of disease severity in patients as well as other regional differences. Furthermore, an Indian study reporting increased occurrence of secondary infections and sepsis, multiorgan dysfunction, and death in diabetic COVID-19 patients also support findings of our study [27]. The data available from other countries showing a higher risk of ICU admission, disease severity, ARDS, and mortality rate in diabetic COVID-19 patients also corroborate with findings of our study [28]. Our lab data showing proinflammatory state and co-existence of vascular complications in diabetic COVID-19 patients further support that DM is a potential risk factor for disease severity and mortality of COVID-19.

Various mechanisms have been implicated in the poor clinical outcome of COVID-19 in diabetic patients. Hyperglycemia causes impairment of the immune system through oxidative stress and impaired functioning of macrophages and neutrophils, resulting in an increased risk of secondary bacterial, viral, and fungal infections, chronic inflammation, and altered cytokine response, leading to cytokine storm, multi-organ dysfunction, and even death [29]. In addition, DM also causes endothelial dysfunction, enhanced platelet aggregation and activation associated with pro-thrombotic hypercoagulable state, and vascular complications such as hypertension, cardiovascular disease, and renal disease [30]. These inter-related pathogenic pathways collectively may lead to the increased disease severity, multi-organ dysfunction, and mortality observed in diabetic COVID-19 patients. In a nutshell, there is a bidirectional relationship between DM and COVID-19. On the one hand, DM and its associated comorbidities and proinflammatory parameters increase the risk of a more severe course of COVID-19 and increase mortality. On the other hand, COVID-19 and its associated hyperinflammation contribute to hyperglycemia by directly or indirectly inducing damage or malfunctioning of pancreatic beta-cells.

Our study provides robust evidence of the direct or indirect role of DM and its associated complications in the worse prognosis, including increased in-hospital mortality of COVID-19 patients. The findings suggest that in clinical practice, hyperglycemic COVID-19 patients with or without a history of DM should be monitored for the development of severe disease for effective management and improved prognosis and outcome of the disease. Our observations also indicate the need for a public health policy for regular blood glucose monitoring of all hospitalized COVID-19 patients, whether diabetic or non-diabetic, to detect the degree of hyperglycemia or COVID-19-induced DM for achieving optimal glycemic control through appropriate anti-diabetic treatment for preventing the risk of severe disease and DM-associated fatal complications. However, despite these strengths, the study also has certain limitations. These limitations mainly include the single-center nature of the study that lacks external validity and generalizability to a broader population, lack for control on confounding variables, and retrospective nature of the study. Another major limitation of the study is the lack of resources for cytokines (IL-6, IL-10, and TNF-α mediating cytokine storm and hyperinflammation), coagulopathy parameters, and lymphocyte subsets to precisely explain the mechanism(s) underlying our observations of this study.

## Conclusions

In conclusion, our study shows a high prevalence of DM in COVID-19 patients and an adverse outcome of DM on the prognosis of the patients, including increased disease severity and in-hospital mortality. We recommend frequent testing of blood glucose levels of hospitalized COVID-19 patients for early detection and treatment of DM to prevent occurrence of severe complications including ARDS, secondary infections, and multiorgan dysfunction and to improve the prognosis and clinical outcome of the disease. The systemic multi-centric studies with inclusion of inflammatory and coagulopathy biomarkers (e.g. IL-6, TNF-α, d-dimer, C1q) need to be conducted at the country level to validate findings of this study and to gain new insights into the prevalence and prognostic impact of DM in Indian COVID-19 patients.
